# Digging gold: keV He^+^ ion interaction with Au

**DOI:** 10.3762/bjnano.4.53

**Published:** 2013-07-24

**Authors:** Vasilisa Veligura, Gregor Hlawacek, Robin P Berkelaar, Raoul van Gastel, Harold J W Zandvliet, Bene Poelsema

**Affiliations:** 1 Physics of Interfaces and Nanomaterials, MESA+ Institute for Nanotechnology, University of Twente, P.O. Box 217, 7500 AE Enschede, The Netherlands

**Keywords:** formation and healing of defects in crystals, helium ion microscopy, ion beam/solid interactions, vacancies in crystals

## Abstract

Helium ion microscopy (HIM) was used to investigate the interaction of a focused He^+^ ion beam with energies of several tens of kiloelectronvolts with metals. HIM is usually applied for the visualization of materials with extreme surface sensitivity and resolution. However, the use of high ion fluences can lead to significant sample modifications. We have characterized the changes caused by a focused He^+^ ion beam at normal incidence to the Au{111} surface as a function of ion fluence and energy. Under the influence of the beam a periodic surface nanopattern develops. The periodicity of the pattern shows a power-law dependence on the ion fluence. Simultaneously, helium implantation occurs. Depending on the fluence and primary energy, porous nanostructures or large blisters form on the sample surface. The growth of the helium bubbles responsible for this effect is discussed.

## Introduction

The helium ion microscope allows the projection of a He^+^ beam of several tens of kiloelectronvolts with a diameter of 0.4 nm [1] onto a sample. This makes HIM an attractive tool for surface patterning and nanofabrication [2–6]. In addition to ultrahigh-resolution imaging, HIM can be utilized for the compositional analysis and crystallographic characterization of samples [7–8]. Since it is a relatively new technique, many questions concerning the interaction of the focused He^+^ beam with matter remain open. As helium ions are light particles, sputtering processes are much less effective with HIM as compared to other focused ion beam (FIB) techniques that typically use gallium ions. Nevertheless, helium ion beam imaging can lead to considerable sample and, in particular surface, modifications. The implantation of He, and the associated possible structural and chemical changes, can create substantial problems in experiments where prolonged imaging or high ion doses are required.

The effect of the He^+^ ions on the target depends as much on the ion beam characteristics as on the properties of the imaged material itself. Existing publications on damage by a focused He^+^ beam mostly concentrate on the interaction of ions with semiconductor materials such as silicon [9–12]. In this paper we investigate the interaction of a He^+^ beam with metals. Previously, the effect of a low-energy He^+^ ion beam on an atomically flat gold surface was observed by scanning tunneling microscopy (STM) [13–14]. Mounds with spacing of a few nanometers were formed. In the current work we have studied the He^+^-ion-induced modifications of crystalline gold samples due to sputtering, helium implantation and defect formation, as a function of ion fluence and energy.

## Experimental

The experiments were performed with an ultrahigh vacuum (UHV) Orion® Plus Helium Ion Microscope from Carl Zeiss NTS [15] at room temperature. As a result of the interaction of the He^+^ beam with the target, secondary electrons (SE), backscattered He (BSHe), and sometimes photons are created. Image acquisition is done by collecting SEs with an Everhardt–Thornley (ET) detector. Due to the nature of the interaction of low-energy ions with matter, the lateral size of the interaction volume in the immediate vicinity of the surface remains extremely small [16–17]. This makes the microscope highly suited for obtaining high-resolution images of the surface topography. An image can further be recorded by simultaneous collection of the backscattered He with a microchannel plate [18]. The microscope is also equipped with a silicon drift detector for the measurement of the backscattered ion energy and a Gatan MonoCL4 Elite detector for the detection of ionoluminescence.

The images were recorded using the ET detector. During the measurements the ion current was kept at 0.7 pA. Brightness and contrast settings were kept constant, and the beam was oriented perpendicular to the surface. Three primary ion energies were used in the experiments: 15, 25 and 35 keV. The images were recorded with 0.68 nm pixel spacing, 2 μs dwell time and 32-line averaging, giving an ion dose per image of 6 × 10^16^ cm^−2^. The chamber base pressure during imaging was in the low 10^−9^ mbar range.

The samples were polycrystalline gold specimens, which are commercially available 200 nm thick Au{111} films on a glass substrate with a Cr interlayer. The textured samples were prepared by hydrogen-flame annealing for 5 min. As a result of the annealing process, grains with an average size of a few micrometers were formed. X-ray diffraction measurements confirmed the primarily {111} textured surface orientation of the grains with a 3.5° wide angular distribution. The grains have random azimuthal orientations. In order to remove carbon contamination, all samples were exposed to a 10 W air plasma for 15 min immediately before loading the samples into the main chamber. After ion implantation the topography of the samples was measured with an Agilent 5100 atomic force microscope (AFM) in intermittent mode. The cantilever was a Mikromasch NSC silicon probe, with a guaranteed tip radius of less than 10 nm, and a typical resonance frequency of 150 kHz. The scan size was 2 × 2 μm^2^.

## Results and Discussion

### Au{111} surface modification

**Figure 1:**
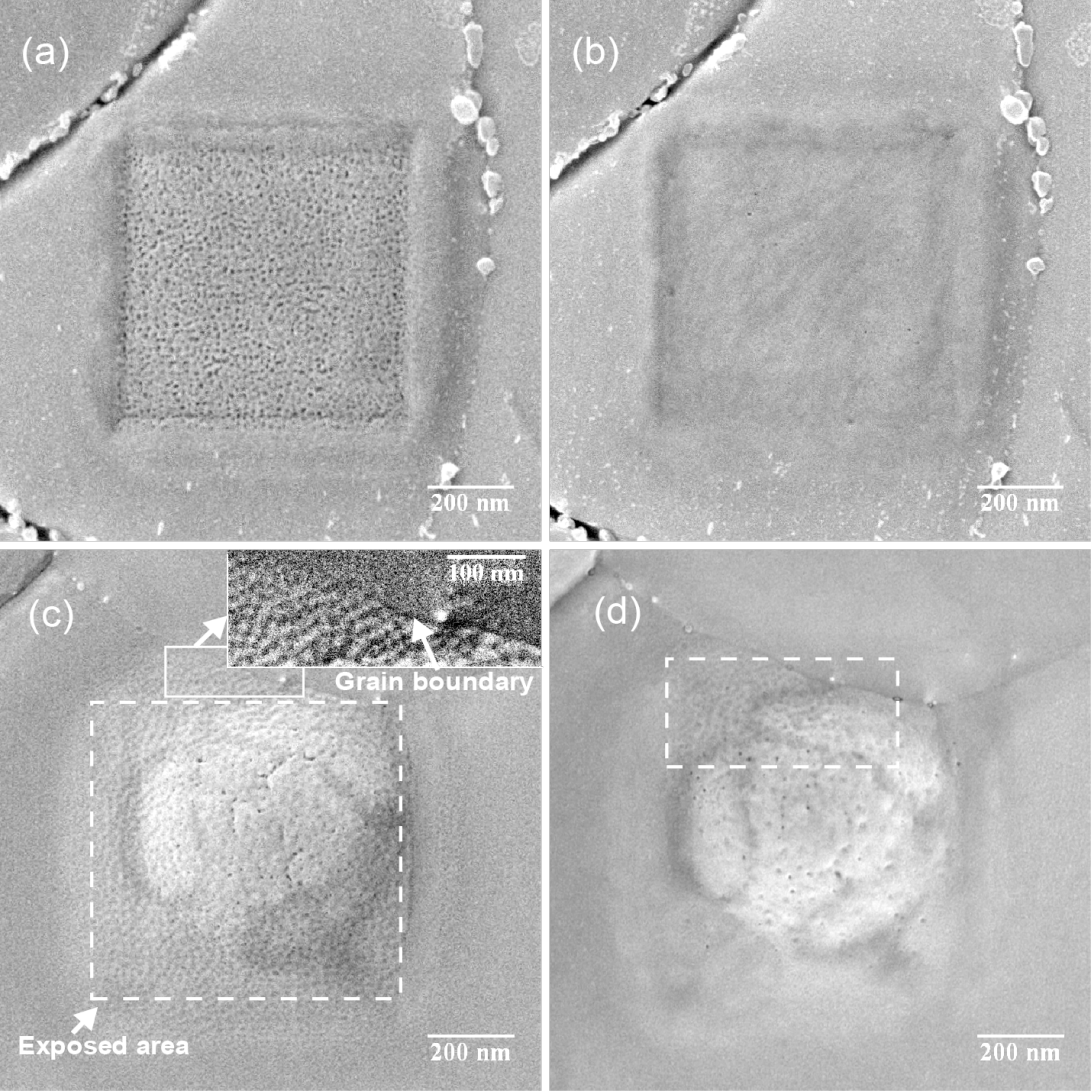
HIM SE image of a Au{111} surface, exposed to a He^+^ beam with a fluence of 8.4 × 10^17^ cm^−2^ at different energies. The field of view (FOV) is 1.25 μm, pixel spacing is 1.5 nm. (a) Porous structure formed by a 15 keV He^+^ beam. (b) The same area as in image (a) after 1.5 months storage under dry atmospheric conditions. The surface has partly self-annealed. (c) Blister formed by a 35 keV beam. The area exposed to the beam is marked by a dashed line. The surface has developed a periodic pattern. The influence of the beam is easily visible outside the marked area as well, but does not extend on the neighboring grain (see inset). (d) The same area as in (c), imaged after 4 months storage under dry atmospheric conditions. The surface of the blister has partly self-annealed, except the marked area in the vicinity of the grain boundary.

We have recorded sequences of images of submicron size to study the evolution of the Au{111} surface under the impact of a focused He^+^ beam as a function of fluence. Ion energies of 15, 25 and 35 keV were used to gauge the influence of the beam energy. The same sample area was exposed to the beam several times with a constant ion dose per scan. The final state of the surface after a fluence of 8.4 × 10^17^ cm^−2^ is shown in Figure 1a and Figure 1c: at 15 keV primary energy a porous structure is formed on the surface (Figure 1a), while in the case of a 35 keV beam a subsurface helium blister is formed (Figure 1c).

**Figure 2:**
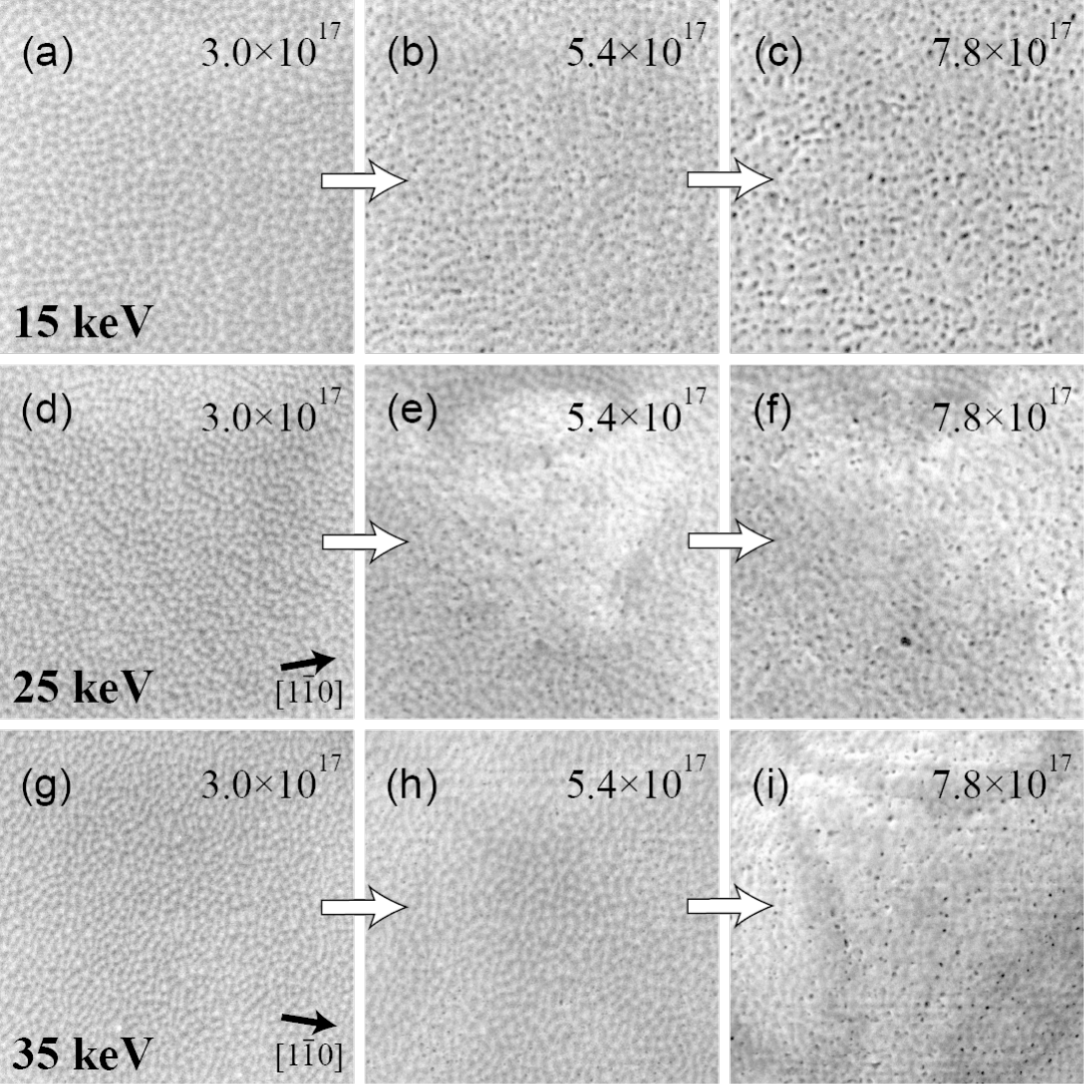
HIM SE images of the pattern that develops on the Au{111} surface as a function of ion fluence. Numbers indicate the ion fluence in helium ions per cm^2^. Arrows in (d) and (g) indicate the azimuthal directions of the grains. The He^+^ beam energies are 15, 25 and 35 keV. FOV is 500 nm, pixel spacing is 0.68 nm.

We emphasize that due to the low background pressure, the present setup does not suffer from the problem of carbon deposition in the imaged area. This is a common problem in conventional non-UHV HIM and scanning electron microscopes (SEM) [15,17,19]. The absence of the carbon layer that is normally present, allows us to obtain detailed information on the surface structure and how it evolves during repeated imaging of the same area. Figure 2 shows several images of the gold surface after exposure to identical ion fluences, but with different primary energies.

Under the influence of the 15 keV beam a regular nanopattern develops. The topographic contrast increases and the surface pattern becomes more pronounced with each subsequent scan of the same area, which indicates an increase of the corrugation of the pattern. Although the feature spacing increases with increasing ion fluence, the shape of the features remains almost unchanged and the features do not coalesce. After a fluence of 3 × 10^17^ cm^−2^ a uniform distribution of holes starts to appear on the surface (see Figure 2a). With a further increase of the fluence the porous structure gets more pronounced (Figure 2b and Figure 2c).

In the case of 25 keV primary ion energy the surface modification initially looks similar to the one at 15 keV (Figure 2d), but at a fluence of 4.8 × 10^17^ cm^−2^ a blister forms, which is shown in Figure 2e. For larger fluences pores start to appear on the surface of the blister (see Figure 2f). A beam with a primary energy of 35 keV initially induces a comparable nanopattern formation (Figure 2g). Higher fluences result in blister formation (Figure 2h) and eventually the formation of a large subsurface helium blister at a fluence of 6 × 10^17^ cm^−2^ (Figure 2i). We also observe some pores on the surface of the blister.

**Figure 3:**
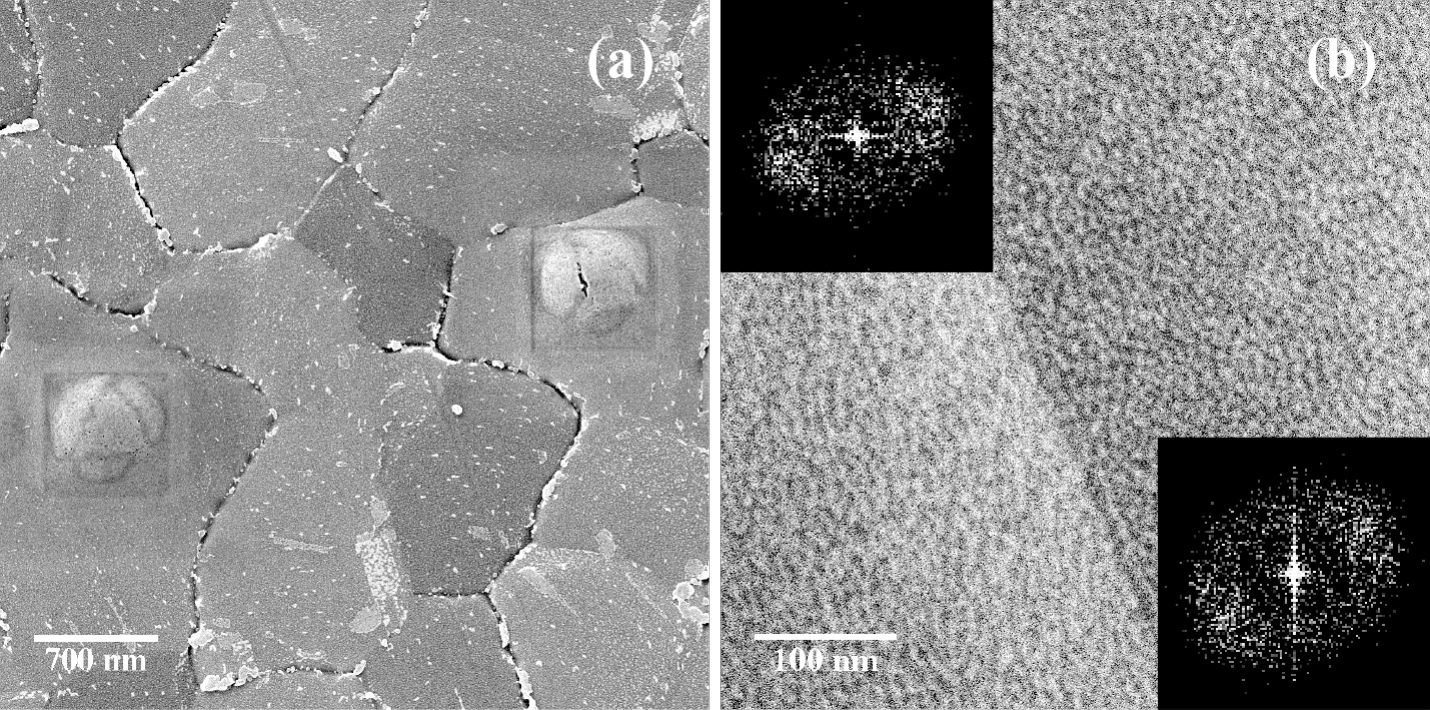
(a) Two blisters created by the 35 keV He^+^ beam on grains with different azimuthal orientation. FOV is 4 μm. (b) HIM SE image of a Au{111} textured polycrystalline film. The insets are 2D FFTs to demonstrate the relation of the patterns to the orientation of the two grains. He^+^ beam energy is 35 keV. FOV is 500 nm.

In Figure 3a two blisters on grains with different azimuthal orientation are shown. Although severe damage has been done to the surface and bulk of the gold grains, their crystalline nature is still evident. The blisters have equilateral triangles on top. The same triangles are also observed in the BSHe images, hinting at the channeling nature of the contrast. We attribute these dark triangles and rings to channeling along the 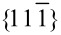 planes of the FCC crystal. The crystalline shell of the blister is bent (see Figure 1c) due to the high internal gas pressure. As a consequence the 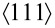 surface vector locally tilts. This leads to a local channeling condition with the 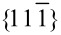 planes along sections of the blister, resulting in the dark bands on the blister surface. The contrast changes with variation of the beam incidence angle, the channeling condition is no longer fulfilled and the dark stripes move or even vanish entirely [8]. The orientations of the sides of the triangles in Figure 2e and Figure 2i help to determine the azimuthal orientations of the grains. Since we used a [111] oriented FCC crystal, the ions are expected to channel along 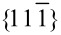 planes [8], which cross the (111) surface along 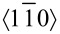 directions. Hence, the sides of the triangles are oriented along 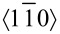, which is indicated with arrows in Figure 2d and Figure 2g.

**Figure 4:**
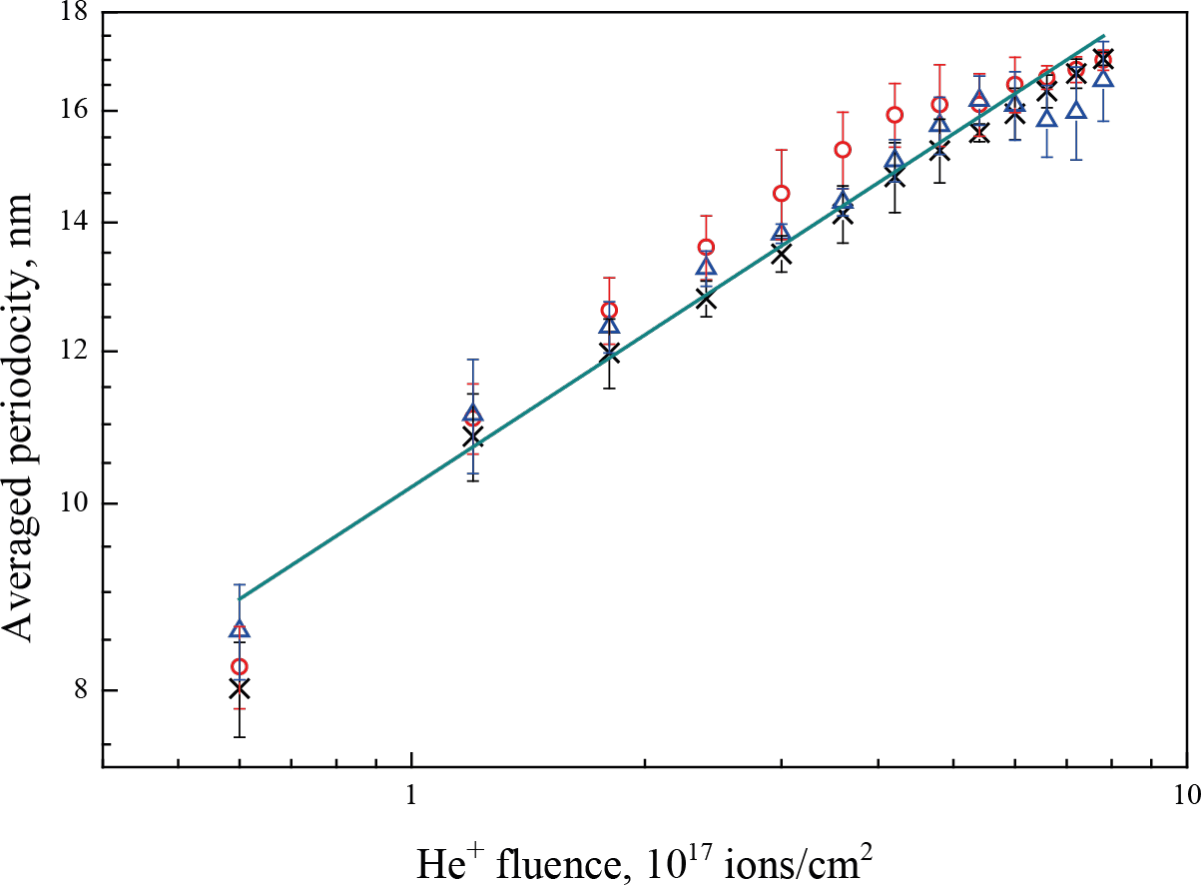
Dependence of the Au{111} average pattern periodicity on helium fluence for 15 keV (red circles), 25 keV (black crosses) and 35 keV (blue triangles) He^+^ beams.

The polycrystalline nature of the samples influences the pattern formation as well. First, the pattern propagation is stopped by grain boundaries as can be seen in the inset in Figure 1c: no pattern or rising of the surface level is observed on the neighboring grain. Second, the pattern orientation depends on the underlying crystal and thus on the orientation of the grain. Figure 3b displays patterns on two neighboring grains. The patterns are rotated relative to each other on the two different grains, as is also visible from the 2D FFT, shown in the insets. The average pattern periodicity was extracted from the images by analyzing 2D autocorrelation functions (ACF). The dependence of the nanopattern periodicity on the He^+^ fluence for different primary energies is shown in Figure 4.

The periodicity does not show a significant energy dependence and increases from 8.3 ± 0.3 nm to a maximum of 16.9 ± 0.4 nm, showing a power-law dependence on the ion fluence with a coarsening exponent of 0.26 ± 0.01. The same scaling with a time factor of 0.27 ± 0.02 was obtained by Ramana Murty et al. [14]. The authors studied the surface morphology of Au{111} during sputtering with 500 eV Ar^+^ ions incident at 45° by real-time X-ray scattering. At temperatures of 20–60 °C they observed the formation of mounds with a characteristic spacing. A similar pattern was also observed on Cu{110}, sputtered by 1 keV Ar^+^ ions at 320 K and normal incidence [20]. The corresponding scaling factor was 0.26 ± 0.02. To a certain extent, sputter erosion and atom deposition are similar processes. A continuum model for the mound formation in molecular beam epitaxy (MBE) predicts a coarsening exponent of 0.25 [21], which is very close to the measured values.

The pattern exhibits a preferential orientation along the 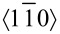 direction (Figure 2d and Figure 2g), as well as the 2D FFTs in Figure 3b). This suggests that the pattern formation is governed by diffusion processes of gold adatoms and surface vacancies. Together with the sputtering processes it leads to surface roughening and the development of a periodic pattern. Although the sputtering rate is low, it cannot be completely neglected. As He^+^ ions impinge on the surface at normal incidence, the sputtering of gold atoms by the direct energy transfer from incoming helium is unlikely. Furthermore, the energy transfer from light helium ions to gold atoms in general is limited because of the unfavorable mass ratio. The sputtering is mainly caused by short-range gold recoils and backscattered helium [22–23]. The presence of the pattern outside of the irradiated area (Figure 1c) is additional evidence of the sputtering by gold recoils. Additionally, the gold interstitials themselves are a source of adatoms on the surface. Gold interstitials are able to travel a few tens of nanometers outside the exposed area, but they cannot cross grain boundaries.

The pattern orientation along a specific crystallographic direction can be explained by considering its formation as a result of the suppression of interlayer diffusion by the step edge or Ehrlich–Schwoebel barrier [14,20,24–28]. The activation energy for vacancy diffusion on Au{111} is much higher than the one for adatoms [29], hence we can suppose that at room temperature adatoms are dominantly responsible for the pattern formation. The presence of a step edge barrier along 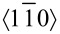 does not allow the adatoms to descend the 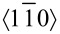 step, and produces a net uphill flow. As a result, mounds are formed along a 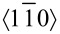 direction. However, one would expect a homogeneous distribution of all three possible pattern orientations due to the symmetry of the {111} surface [28]. The out-of-plane orientation of the grains has some angular distribution. Hence, the surfaces are not atomically flat and have a local miscut. The step edges run in one of the three high-symmetry directions that become preferential for the pattern orientation on any one grain.

The exposed areas were imaged again after several weeks. Samples were stored under dry ambient conditions between the experiments. As can be seen in Figure 1, the surface has a tendency to self-anneal over time. In Figure 1b the same area as in Figure 1a, which was initially exposed to a 15 keV He^+^ beam, is presented, but after six weeks. The blister, formed by the 35 keV beam and presented in Figure 1c, was imaged again after 16 weeks. The image is shown in Figure 1d. In both cases the pattern has almost completely vanished, except in areas close to the grain boundary (inset in Figure 1c), which apparently acts as an efficient sink for adatoms and interstitials. Thus it hinders the smoothing of the surface in the vicinity. The surface is smoothed, but after a few repetitive scans, the pores, hidden deeper in the substrate, open again. The blister shell self-anneals over time, indicating a possibility to heal the defects. That process can be enhanced by in situ heating of a sample during ion bombardment.

We mention, that the surface modification depends not only on the final fluence, but also on the speed at which it was generated. With an increase of the dose per scan, the modifications occur more swiftly and are more severe.

### Helium implantation

Helium implantation occurs during sample irradiation. Since HIM SE images do not contain height information, we have used AFM to directly measure the volume that is occupied by the implanted helium. As a result of the low background pressure of hydrocarbons in the UHV HIM we can exclude false volume estimations due to carbon contamination.

**Figure 5:**
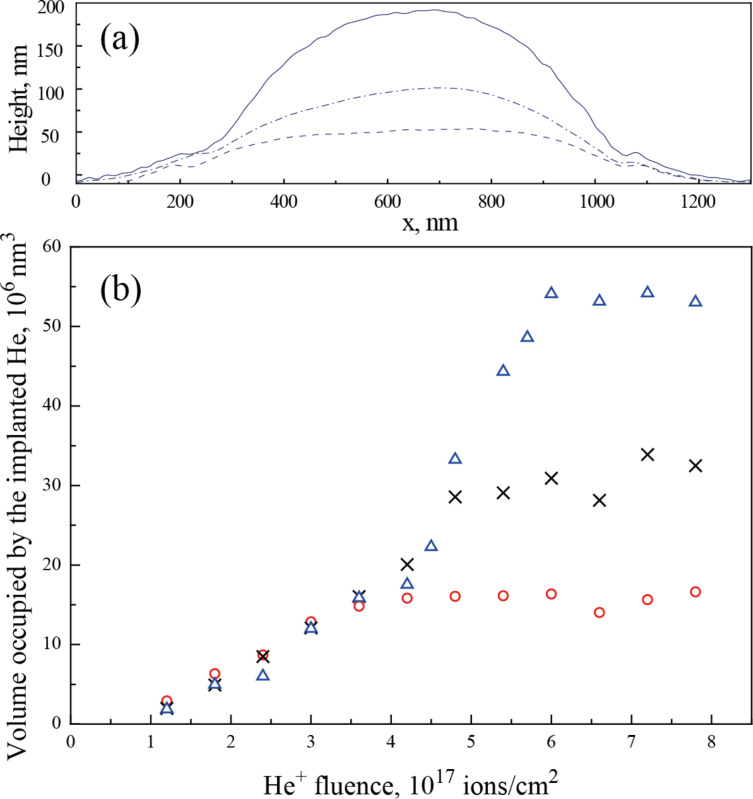
(a) Surface profiles after different ion fluences delivered by a 35 keV beam. The surface is evenly raised after 4.2 × 10^17^ cm^−2^ (dashed line). The dash-dotted line shows the profile of a blister that starts to form after a fluence of 4.8 × 10^17^ cm^−2^. After 6 × 10^17^ cm^−2^, the blister develops a stable shape (solid line). (b) Volume occupied by the implanted helium as a function of helium fluence. The beam primary energies are 15 keV (red circles), 25 keV (black crosses) and 35 keV (blue triangles).

The change of the surface profile with ion fluence for a primary energy of 35 keV is shown in Figure 5a. After a fluence of 4.2 × 10^17^ cm^−2^, the surface is still comparatively flat (dashed line), but already for a slightly larger fluence a subsurface blister develops. The profile of a growing blister at 4.8 × 10^17^ cm^−2^ is shown by the dash–dotted line. At 6 × 10^17^ cm^−2^ a blister with a stable shape has developed (solid line).

After an initial dose of 6 × 10^16^ cm^−2^ the exposed area is eroded by 1.0–1.5 nm with respect to the nonirradiated surface. This is the result of sputtering of a few gold layers. The signature of this sputtering-related indentation remains discernible in all subsequent images. After doubling the dose to 1.2 × 10^17^ cm^−2^, helium implantation has a noticeable effect: the surface of the exposed square and also the unexposed area adjacent to it, starts to rise with increasing ion fluence. The influence of the helium implantation extends as far as 144 ± 12 nm (15 keV), 162 ± 6 nm (25 keV) and 181 ± 7 nm (35 keV) away from the exposed area. In Figure 5b the increase of blister volume due to helium implantation is presented as a function of ion fluence. The volume depends linearly on the fluence up to 4.2 × 10^17^ cm^−2^. After this total dose, the volume occupied by ions implanted at 15 keV stagnates at (15.8 ± 0.3) × 10^6^ nm^3^. In the case of 35 keV ions, after a fluence of 4.2 × 10^17^ cm^−2^ a more rapid expansion begins. Later, when the fluence reaches 6 × 10^17^ cm^−2^, the volume saturates at (54.2 ± 0.4) × 10^6^ nm^3^. For the energy of 25 keV the rapid expansion sets in at the same fluence, but saturates at an intermediate level of (30.5 ± 0.9) × 10^6^ nm^3^.

In the review by Donnelly [30], surface swelling of several materials (Er, Nb and Ni) under helium irradiation is compared. The general trend of the expansion is similar to the one described in this work. The initial linear expansion was found to be energy-independent as well. In the work of Terreault et al. [31], the authors studied helium trapping in Cu, which has similar physical properties to Au. In this case blistering was observed after a fluence of 4.0 × 10^17^ cm^−2^.

As is seen from the Figure 5b, there is a negative volume offset, which is attributed to two effects. First, sputtering of the surface will result in material loss. Secondly, at low ion fluences helium ions can occupy existing crystal defects and interatomic positions without causing a substantial volume increase. The subsequent fluence increase leads to the creation of helium nanobubbles in the bulk gold. The formation of voids in metals due to He^+^ ion bombardment is a well-known phenomenon [32–34]. After entering the crystal, an energetic He^+^ ion creates vacancy–interstitial pairs. These vacancies can aggregate into bigger voids. Since helium is hardly solvable in metals, it is effectively trapped at open-volume defects and has a tendency to agglomerate into nanosized bubbles [35–36]. That leads to deformations, which cause the initial linear volume increase in the graph in Figure 5b. At these fluences (up to 4.2 × 10^17^ cm^−2^) the volume change does not depend on the primary energy of the implanted ions.

As more helium ions are implanted, the cavities expand. The helium nanobubbles are highly over-pressurized. Up to a certain bubble size the excess pressure is relieved by loop punching. This bubble growth mechanism was first suggested by Greenwood et al. [37] and later on discussed by Evans [38]. As bubbles grow, several neighboring bubbles eventually create enough local stress to create a crack in the crystal and coalesce. At higher fluences the different stopping powers of gold and (high pressure) helium become relevant. At low energies helium is implanted in a near-surface region. This near-surface helium volume is an effective stopping material for more helium. As a result, a rapid expansion sets in until the bubble reaches the surface. The above described porous structure develops (Figure 1a). At higher energies these processes occur deeper in the material and more helium is incorporated, and as a result a blister develops. The blister formation mechanism by interbubble fracture, has been suggested by Evans [39]. However, also at these high energies helium will start to leak to the surface and the blister growth saturates. The steep part of the graph at 35 keV in Figure 5a corresponds to the blister formation and growth process. At 25 keV this stage of the damage development was not resolved and only the volume of the already formed blister was measured.

We have made rough estimations of the pressure in the nanobubbles, and the pressure in the final blister at 35 keV. Not all of the incident helium is trapped in the bubbles: a part of it is backscattered, and some diffuses into the bulk or out of the material. SRIM-2011 [40] has been used to assess the percentage of backscattered helium. A gold slab with a thickness of 200 nm and 10^5^ ions have been used in the calculations. According to these simulations 16% of the incident helium is backscattered at 35 keV. Attributing 4% to other loss mechanisms we used 80% of the fluence for our further calculations. Two approaches were used for the pressure estimation. In the first approach, the pressure was calculated using the virial equation of state:

[1]
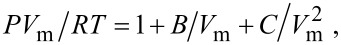


where *P* and *T* are the helium pressure and temperature respectively, *V*_m_ is the helium molar volume, *R* is the universal gas constant, *B* and *C* are the second and third virial coefficients. The values of *B* and *C* for He at room temperature were taken from [41] and [42]. This gives a lower estimate of 2.1 GPa for the pressure in the nanobubbles just before the start of the rapid expansion. Another assessment was done by applying a relation used by Evans [38], which is based on the work of Rowlinson [43]:

[2]
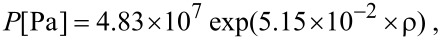


where ρ is the helium density in units of atoms/nm^3^. In this case the calculated pressure is 6.1 GPa. Please note that these two estimates only give an idea of the order of magnitude of the He pressure inside the nanobubbles. As the bubbles grow in size the material cannot support such high pressures, and the bubbles merge. In the case of the final blister grown with a primary energy of 35 keV, both models yield similar values of 437 MPa and 442 MPa, respectively.

## Conclusion

Exposure to high He^+^ ion fluences has a dramatic influence on a crystalline sample, which strongly depends on the energy of the incident beam. Sample modifications are mainly caused by helium implantation producing surface deformations. After the initial formation of nanobubbles filled with helium in the gigapascal pressure range, different scenarios evolve. At low energies the bubbles quickly reach the surface and release the helium, and a sponge-like surface develops. At high energies, the initial nanobubbles form deeper in the material due to the greater range of the helium ions. Consequently, bubble coalescence leads to the formation of a large blister that continues to grow. The final size before the shell leaks depends on the primary energy and thus the implantation depth.

During irradiation with He^+^ ions at normal beam incidence also a periodic nanopattern develops on the surface at room temperature. The pattern is oriented along the 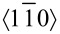 direction and its periodicity scales with the ion fluence with a coarsening exponent of 0.26 ± 0.01. The observed features do not coalesce and preserve their shape. An important observation is that the beam influences not only those areas that are directly irradiated by the beam, but also the neighboring regions.
